# Spatial and Seasonal Changes in Microbial Community of *Hynobius amjiensis* Breeding Pools in a *Sphagnum*-Dominated Peatland

**DOI:** 10.3390/microorganisms12071344

**Published:** 2024-06-30

**Authors:** Meng-Jie Yu, Xian-Ting Wang, Ting Wang, Wei-Quan Huang, Ze-Dong Lang, Jia-Peng Wang, Yu-Huan Wu

**Affiliations:** 1 College of Life and Environmental Sciences, Hangzhou Normal University, Hangzhou 311121, China; mengjie.yu@hznu.edu.cn (M.-J.Y.); wangting716@126.com (T.W.); 2022111010097@stu.hznu.edu.cn (W.-Q.H.); 2 Zhejiang Hynobius amjiensis Nature Reserve Management Office, Huzhou 313300, China; 3 School of Information Science and Technology, Hangzhou Normal University, Hangzhou 311121, China; 4 Zhejiang Provincial Key Laboratory of Urban Wetlands and Regional Change, Hangzhou 311121, China

**Keywords:** peatland, egg sacs, water quality, microbial community, spatial heterogeneity, seasonal variation

## Abstract

Peatlands deliver a variety of beneficial ecosystem services, particularly serving as habitats for a diverse array of species. *Hynobius amjiensis* is a critically endangered amphibian initially discovered in a *Sphagnum*-dominated peatland in Anji, China. The unique habitat requirements of *H. amjiensis* make it highly vulnerable to environmental changes. Here, we investigated the different breeding pools of *H. amjiensis* in the *Sphagnum*-dominated peatland (the type locality) for a one-year period to evaluate the interactions among the egg sacs present, water quality, and microbial communities (16S and 18S rRNA gene amplicon). The numbers of egg sacs were higher in the breeding pools located at the marginal area than those at the core area of the peatland. Similarly, the α-diversity of bacteria, fungi, and protists were lower in the core region compared to those at the edge of the peatland, perhaps due to water eutrophication. The microbial communities and water quality differed significantly among breeding pools and sampling months. The simpler microbial networks of the breeding pools in the core wetland may impact the numbers and health of the egg sacs. This study contributes to a better understanding of the effect of water quality on biodiversity in peatlands, and it can also guide regulations for wetland conservation and the protection of endangered species.

## 1. Introduction

Peatlands provide globally crucial ecosystem functions such as biodiversity support, nutrient cycling, and water and climate regulation. They consist of habitat mosaics containing plant species that form peat [1]. *Sphagnum* mosses (peat mosses) are regarded as peatland engineers since most of the accumulated peat in peatlands is derived from *Sphagnum* mosses [2,3]. The unique acidic, waterlogged, and oxygen-limited conditions serve as habitats for specific animals [4]. However, global warming, drought, and land use change, coupled with other human activities, have led to the severe degradation of peatland, with consequent effects on biodiversity [5].

*Hynobius amjiensis* is a critically endangered amphibian in the family Hynobiidae, endemic to eastern China. It was firstly discovered in the Qianmutian *Sphagnum*-dominated wetland (subtropical peatland) in Anji, Zhejiang Province (the type locality) by Chinese herpetologist Huiqing Gu [6]. The salamander typically lays its eggs in small pools with clear, cold, and weakly acidic water during the winter season, from December to March [7]. The pools must be surrounded by dense peat moss, which provides shelter and protection for both adult salamander and larvae [8,9]. *H. amjiensis* is classified as an endangered species according to the IUCN Red List of Threatened Species Class I Wildlife species [10]. Chen et al. (2016) investigated the breeding ecology of *H. amjiensis* over an eight-year period (2007–2014) and observed only 20–35 egg sacs each year, indicating a very limited number of breeding females in the wetland [8].

The specific habitat requirements of *Hynobius amjiensis* make it extremely vulnerable to environmental changes and habitat shrinkage [11]. The Qianmutian *Sphagnum*-dominated wetland is an intermontane basin wetland, primarily receiving its water from surface and groundwater runoff from upland areas. In recent decades, the water supply has diminished due to global warming and drought, leading to the invasion of vascular plants and a consequent shrinkage of the *Sphagnum*-dominated wetland [4]. The vascular plants and mosses provided different root exudates and plant litter as C source for different microbial communities. Meanwhile, the wetland has undergone gradual eutrophication, mainly through runoff and nutrient deposition, modifying or changing biological communities. Microbial communities play a crucial role in nutrient cycling, ecological regulation, and the maintenance of aquatic ecosystem health [12,13]. Identifying and quantifying the ecological processes and factors that regulate the assembly of microbial communities is a central focus in microbial ecology [14,15]. Previous studies reported that microbial composition in aquatic ecosystems is significantly affected by various environmental factors, including water temperature [16], pH [13], and N, P, and other nutrients [14]. Thus, microorganisms are highly susceptible to environmental changes and can serve as ecological indicators for nutrient inflow and hydrological fluctuation. It is necessary to investigate comprehensively the correlation between water quality and microbial communities of breeding pools of *H. amjiensis*, and to what extent microbial communities can reflect the water quality of the breeding pools.

Herein, six scattered breeding pools were selected to explore the spatial and seasonal variations in the conditions of egg sacs, water quality, and microbial communities in Qianmutian *Sphagnum*-dominated wetland. Specific objectives were (1) to investigate the number and conditions of egg sacs of *Hynobius amjiensis* in different breeding pools; (2) to assess the spatial and seasonal heterogeneity of water properties and microbial communities in the breeding pools; and (3) to examine the impact of water quality and microbial traits on the egg sacs.

## 2. Materials and Methods

### 2.1. Study Area

The Qianmutian *Sphagnum*-dominated wetland (30°23′ N, 119°26′ E) is the core habitat for *Hynobius amjiensis* in Zhejiang *Hynobius amjiensis* National Nature Reserve, which is located in Anji County (Zhejiang Province, China). Detailed information about the wetland is presented in Yu et al. (2023) [4]. Briefly, the study area has a subtropical monsoon climate with an average annual rainfall of 1870 mm. The temperatures range from 2.6 °C to 28 °C [17]. The wetland is at elevations between 1300–1600 m and primarily receives its water supply from the surrounding upland regions.

**Figure 1:**
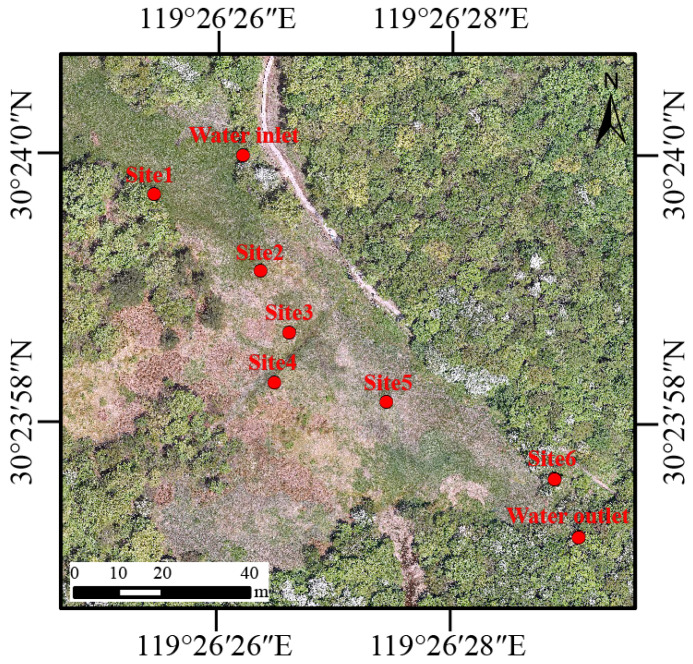
Sampling sites including one water inlet, six breeding pools (Site 1–6), and one water outlet within the wetland area in Zhejiang *Hynobius amjiensis* National Nature Reserve, China.

There are six pools (Site 1–6) designated as breeding habitats for *Hynobius amjiensis* in Qianmutian *Sphagnum*-dominated wetland (Figure 1). The area and depth of the pools are shown in Table S1. *H. amjiensis* seems to breed in pools surrounded by *Sphagnum* moss from mid-February to early April [8]. We counted the number of egg sacs in the six breeding pools in early March.

### 2.2. Water Sampling and Analysis

In 2022, water samples were collected each month (except February) from one water inlet, six breeding pools (Site 1–6), and one water outlet within the wetland area (Figure 1). Sampling was suspended in February due to the closure of the National Nature Reserve, which was attributed to inclement weather and the Chinese Spring Festival holiday. Every month, in situ measurements of water temperature, pH, electrical conductivity (EC), and dissolved oxygen (DO) were performed on each sampling site (water inlet and outlet, Site 1–6) with a multi-probe (SHINWA 72716, SHINWA, Amakusa, Japan). The turbidity (as Nephelometric Turbidity Units, NTU) was determined with a portable turbidity meter (HACH 2100Q, Loveland, CO, USA). In addition, 2 L of water was taken from the surface (10 to 20 cm) at each sampling site, and then prefiltered with 180-μm nylon mesh to reduce impurities. In total, we obtained 88 water samples (8 sampling sites × 11 months). Each water sample was separated into two parts. 1 L of water was used to determine the total nitrogen (TN), total phosphorus (TP), chemical oxygen demand by potassium permanganate oxidation (COD_Mn_), and chlorophyll *a* (Chl*a*) were determined. TN was determined using the alkaline potassium persulfate digestion-UV spectrophotometric method. TP was measured using the ammonium molybdate–UV spectrophotometry method. The acidic potassium permanganate oxidation method was adopted to measure COD_Mn_. A spectrophotometric method was used to measure Chl*a*. The above data for water quality is provided by Zhejiang *Hynobius amjiensis* Nature Reserve Management Office. Water (1 L) was sequentially filtered through a 0.22-μm polycarbonate membrane (Xingya, China) to collect microbes in the water and extract microbial DNA. The membrane that contained water microbes was immediately placed in sterile centrifuge tubes and stored at −20 °C until DNA extraction.

### 2.3. DNA Extraction, Illumina High-Throughput Sequencing

Microbial DNA was extracted using the cetyltrimethylammonium bromide (CTAB)-based method. The quality and quantity of the extracted DNA were determined using a Nanodrop spectrophotometer (Thermo Fisher Scientific, Waltham, MA, USA) and agarose gel electrophoresis. The V4–V5 region of the bacterial 16S rRNA gene was amplified with 515F (5′-GTGCCAGCMGCCGCGGTAA-3′) and 907R (5′-CCGTCAATTCCTTTGAGTTT-3′) primers. The V4 region of 18S rRNA gene was amplified with TAReuk454FWD1 (5′-CCAGCASCYGCGGTAATTCC-3′) and TAReukREV3 (5′-ACTTTCGTTCTTGATYRA-3′) primers. The detailed amplification, library preparation, and high-throughput sequencing processes are presented in Supplementary Material and were completed by Shanghai Personal Biotechnology Co., Ltd., Shanghai, China. Sequences were submitted to the Genome Sequence Archive (GSA) database under accession number CRA016685 and CRA016718.

The paired-end raw sequence data were demultiplexed using the “demux” plugin and the primers were cut using the “cutadapt” plugin with QIIME2 platform [18]. Then, the sequences were quality-filtered, denoised, merged, and chimeras were removed using the “DADA2” plugin [19]. Non-singleton amplicon sequence variants (ASVs) were aligned with “mafft” [20]. Taxonomy was assigned to bacterial, fungal, and protist ASVs using the classify-sklearn naive Bayes taxonomy classifier in “feature-classifier” plugin [21] based on SILVA-132 database [22], NCBI database [23], and the PR2 (Protist Ribosomal Reference database) [24], respectively. All resulting sequences were rarefied at a minimum number of sequences per sample (44,465 and 39,865 for 16S and 18S, respectively) for downstream analysis. ASVs affiliated with *Metazoa*, *Opisthokonta*, *Rhodophyta*, *Streptophyta* and other unassigned species in the protist ASV table were filtered prior to analysis.

### 2.4. Microbial Network Creation

Eleven water samples from eleven months for each breeding pool were included to create co-occurrence networks. We removed the ASVs present in less than 7 months and with total abundances lower than 12 reads across all samples. A valid co-occurrence correlation was assigned between bacterial, fungal, and protist ASVs if the Spearman’s correlation coefficient (r) was greater than 0.7 with a *p* value < 0.01. The *p* values were adjusted by multiple testing corrections using the Benjamini–Hochberg false discovery rate (FDR) controlling procedure [25]. The construction of the 8 networks was made using “Hmisc” [26] and “igraph” packages [27] in R (v. 4.2.1), and the figures were plotted by Gephi (v. 0.10.1) to calculate topological properties [28]. The degree, modularity, betweenness centrality, closeness centrality, clustering coefficient, and path length were also calculated by Gephi.

Connectivity of each node was determined by two parameters: within-module connectivity (*Zi*) and among-module connectivity (*Pi*). All nodes were divided into four subcategories based on *Zi*-*Pi*: network hubs (nodes that highly connected with other nodes within the entire network, *Zi* > 2.5 and *Pi* > 0.62), module hubs (nodes that highly connected with other nodes within modules, *Zi* > 2.5 and *Pi* ≤ 0.62), connectors (nodes that have connections to several modules, *Pi* > 0.62), and peripherals (nodes that have few outside connections, *Zi* < 2.5 and *Pi* ≤ 0.62).

### 2.5. Statistical Analysis

Alpha-diversity indexes (Chao1 and Shannon–Wiener) were calculated for microbial communities. Non-metric multidimensional scaling (NMDS) analysis of soil microbial communities was performed based on Bray–Curtis distances. The Mantel test was used to analyze the correlations between water characteristics and microbial community structure. Chao1, Shannon–Wiener, NMDS, Mantel test, and principal component analysis (PCA) were all conducted with “vegan” package [29]. The relative abundances of microbial taxa were visualized with the R package “circlize” [30]. The correlation between microbial taxa and water properties was calculated with the R package “psych” [31]. The bubble plots, bar plots, and box plots were created with “ggplot2” [32] in R. Differences among breeding pools and months were tested using one-way analysis of variance with Tukey’s honestly significant difference (HSD) test.

## 3. Results

### 3.1. Egg Sacs and Water Quality in Breeding Pools

**Figure 2:**
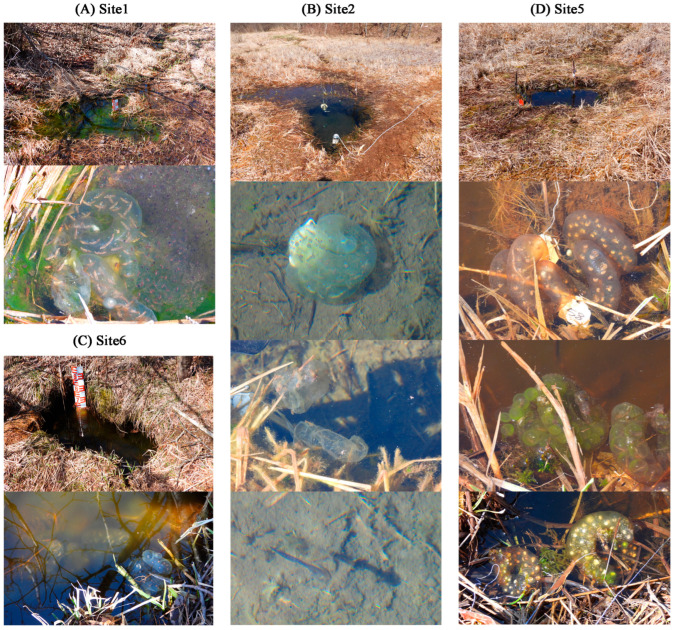
Egg sacs of *Hynobius amjiensis* in the breeding pools (Site 1, 2, 5, and 6).

In Site 1, 2, 5, and 6, there were 1, 3, 14, and 2 egg sacs of *Hynobius amjiensis* observed, respectively (Figure 2 and Table S1). Most egg sacs were laid approximately 10 cm deep without being attached to the substrate. Egg sacs in Site 1, 2, and 6 developed well. Three hatchlings were observed in Site 2. However, some embryos within the egg sacs died at an early developmental stage in Site 5. No egg sacs observed in Site 3 and 4.

**Figure 3:**
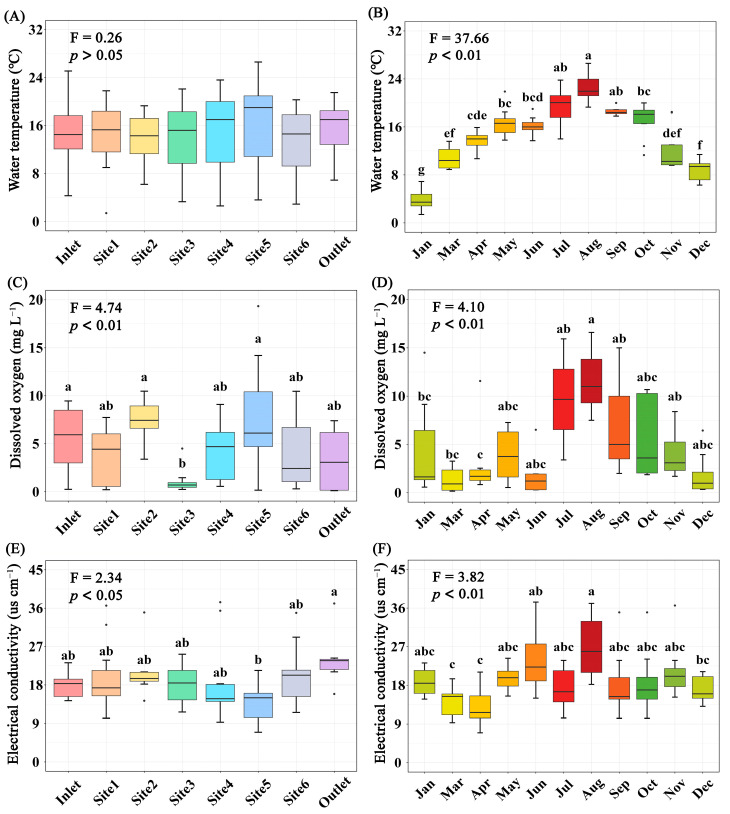
Variations of water temperature (**A**,**B**), dissolved oxygen (**C**,**D**), and electrical conductivity (**E**,**F**) in water of different sampling sites (**A**,**C**,**E**) or months (**B**,**D**,**F**). Boxes are bounded on the first and third quartiles, divided by median lines. Black dots represent outlying data. Boxes with different lower-case letters are significantly different (*p* < 0.05) using Tukey HSD post-doc test.

**Figure 4:**
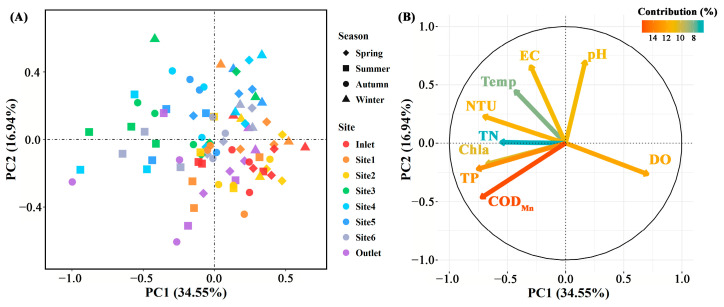
The principal component analysis of physicochemical parameters in water samples (**A**) and their contributions to the variance (**B**) in different sampling sites (*n* = 88). Spring: March–May; Summer: June–August; Autumn: September–November; Winter: January and December. EC: electric conductivity; Temp: temperature; DO: dissolved oxygen; NTU: turbidity; TN: total N; TP: total P; COD_Mn_: chemical oxygen demand by potassium permanganate oxidation; Chl*a*: chlorophyll *a*.

The concentration of total P, COD_Mn_, DO (*p* < 0.01), and EC (*p* < 0.05) differed significantly among breeding pools, while the water temperature, concentration of total N and Chl*a* (*p* > 0.05) did not (Figure 3 and Figure S1). Specifically, the total P and COD_Mn_ in the breeding pools at the center of the wetland were higher than those at the edge of the wetland. The highest water temperature, contents of total N, P, COD_Mn_, Chl*a,* and DO were found in summer, especially in June, July, and August (Figure 3 and Figure S2). PCA analysis was performed to investigate the overall structural variations of water characteristics with different sampling sites and months (*n* = 88, Figure 4). Clear separation of breeding pools at the center (Site 3–5) from marginal areas (water inlet, Site 1, 2) along both PC1 and PC2 highlighted the categories of water quality with close association, such as total P, COD_Mn_, Chl*a,* and pH (Figure 4B).

### 3.2. Microbial Diversity and Community Structure

**Figure 5:**
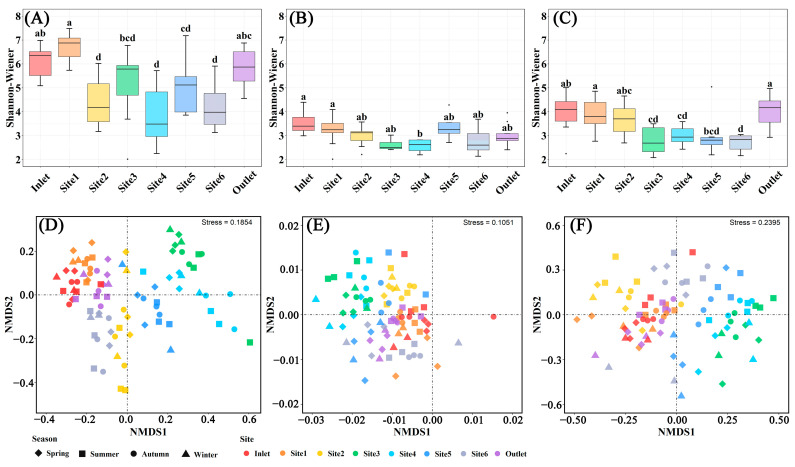
Microbial structures in water samples of different breeding pools with four seasons. Boxplots of α-diversity as revealed by Shannon–Wiener index for bacterial (**A**), fungal (**B**), and protist (**C**) communities in the water samples of different breeding pools with all sampling months. Boxes are bounded on the first and third quartiles, divided by median lines. Black dots represent outlying data. Boxes with different lower-case letters are significantly different (*p* < 0.05) using Tukey HSD post-doc test. Non-metric multidimensional scaling ordination plot (NMDS) based on Bray–Curtis dissimilarity showing the change of soil bacterial (**D**), fungal (**E**), and protist (**F**) community structures. Spring: March–May; Summer: June–August; Autumn: September–November; Winter: January and December.

The microbial α-diversity differed among sampling sites, while the sampling months did not have a significant impact on microbial diversity (Figure 5, Figures S3 and S4). The microbial Chao1 and Shannon–Wiener indices in the breeding pools at the center of the wetland were lower than those at the edge of the wetland (Figure 5A–C and Figure S3), contrary to the water chemical parameters including total P and COD_Mn_ (Figure 3). The lowest bacterial and fungal diversity was found in Site 4 (Shannon–Wiener: 3.87 for bacteria, 1.80 for fungi), and the lowest protist diversity was found in Site 6 (Shannon–Wiener: 2.42 for protist). Non-significant differentiation of microbial α-diversity among different months was recorded due to the variations among sampling sites (Figure S4).

NMDS ordination revealed significant differences in the structures of bacterial, fungal, and protist communities among various water sampling sites (Figure 5D–F). Specifically, distinct clustering of coordinates was observed among sampling sites located at the center of the wetland (Site 2–5), indicating substantial variations in bacterial and protist community structures. The communities of bacteria and protists showed similarity among the inlet and outlet of the wetland and Site 1. Although the impact of different sampling sites on the fungal community structure was relatively minor compared to bacteria, differences were still recorded between the center and edge of the wetland. Nevertheless, the disparities in microbial community structure among different seasons were relatively negligible.

### 3.3. Microbial Community Composition

**Figure 6:**
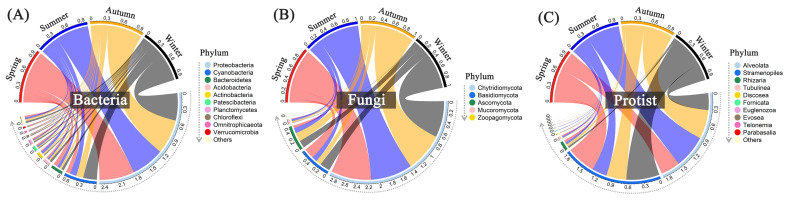
Relative abundances of dominant bacteria (**A**), fungi (**B**), and protists (**C**) among different seasons with all sampling sites at the phylum level. Spring: March–May; Summer: June–August; Autumn: September–November; Winter: January and December.

Proteobacteria and Cyanobacteria, the predominant bacterial phyla in each group, account for more than 75% of the total relative abundance across all sampling sites or months (Figure 6 and Figure S5). The relative abundance of Cyanobacteria was much higher in winter (26%) than those in other seasons, contrary to Proteobacteria, while the relative abundance of Actinobacteria was higher in summer (5%), contrary to Bacteroidetes. All water samples shared a similar composition of fungal communities, comprised of three major phyla: Chytridiomycota, Basidiomycota, and Ascomycota (Figure 6 and Figure S5). Both sampling sites and months affected the relative abundances of protists. Alveolata exhibited higher abundance levels in Site 2–5, especially from May to November, whereas Stramenopiles dominated in Inlet, Site 1, 6, and Outlet, especially from January to April.

### 3.4. Correlation between Microbial Community and Water Properties

A Mantel test revealed that water properties significantly affected bacterial community structure, including water temperature, Chl*a*, total P, and COD_Mn_ (*p* < 0.01). However, the fungal and protist communities were only significantly affected by water temperature (*p* < 0.01) (Table 1).

**Figure 7:**
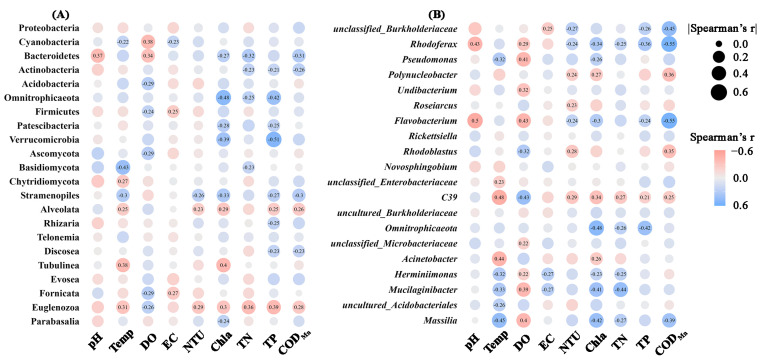
Significant Pearson correlations (*p* < 0.05) between physicochemical properties and relative abundances of microbial phyla (**A**) or bacterial genera (**B**). Circles without numbers represent non-significant correlations (*p* > 0.05). The circles size and color represent the magnitude and direction of correlation coefficients. EC: electric conductivity; Temp: temperature; DO: dissolved oxygen; NTU: turbidity; TN: total N; TP: total P; COD_Mn_: chemical oxygen demand by potassium permanganate oxidation; Chl*a*: chlorophyll *a*.

The Pearson correlation coefficients were applied to evaluate the correlations between water physicochemical variables and the abundances of the microbial groups (Figure 7). Significant correlations were noticed between the majority of the properties and the microbial abundances. Specifically, at the phylum level, DO, Chl*a*, total P emerged as primary environmental factors influencing bacterial phyla abundances. Interestingly, higher concentrations of total N, total P, Chl*a,* and COD_Mn_ were associated with increased abundances of most bacterial phyla. Additionally, protist groups showed significant correlations with environmental factors, particularly among Stramenopiles, Alveolata, and Euglenozoa. At the bacterial genera level, temperature, DO, and Chl*a* showed the highest number of correlations with the top 20 genera. Specifically, the abundances of *Rhodoferax*, *Pseudomonas*, *Flavobacterium*, *Herminiimonas*, *Mucilaginibacter,* and *Massilia* were significantly negatively correlated with DO, but positively correlated with Chl*a*.

### 3.5. Topological Properties of Microbial Networks

**Figure 8:**
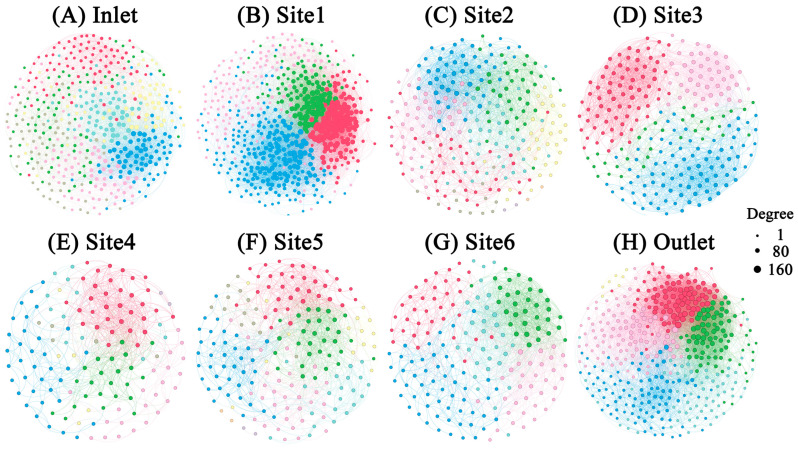
The microbial co-occurrence networks of water samples from different sampling sites. Modules are labelled with different colors in the respective networks. The nodes within one module shared the same color. The size of each node is proportional to the number of links (that is, degree). Node pairs with Spearman coefficients > 0.7 were connected.

To investigate the co-occurrence patterns of microbial groups in breeding pools, microbial networks were modulated for eight sampling sites (Figure 8). Multiple network topological metrics revealed markedly different structures for the different sampling sites (Table 2). The breeding pools at the edge of the wetland formed greater networks with more nodes and links than those at the center of the wetland. The network with the fewest nodes and links was found in Site 4 (nodes: 138; links: 638), while the most complex network was observed in Site 1 (nodes: 594; links: 15270). Meanwhile, the networks within Site 1 and the water outlet contained higher numbers of links which contributed to the higher density of connections, but less negative links between nodes than others. A module was defined as a group of ASVs that were well connected among one another but less linked with the ASVs belonging to other modules. Distinct modules were observed between breeding pools. A total of seven modules with more than five nodes were obtained for the networks of water inlet, Site 2 and 5, while the networks of Site 1 and 3 only had four modules with more than five nodes.

Most of the putative keystone taxa were identified as bacteria (Table S2). Only two protist ASVs (Stramenopiles and Alveolata) classified as module hubs in the water inlet network. No module hubs were found in Site 3 and 4. The networks of the water inlet and Site 1 had the largest numbers of connectors, followed by water outlet, Site 2 and 5.

## 4. Discussion

### 4.1. Seasonal and Spatial Variation of Water Quality in Breeding Pools

Water temperature is an important factor in determining the primary productivity of natural water bodies, affecting their physicochemical properties and biological activities [33]. The elevated temperature in summer catalyzed eutrophication by creating conditions that increased nutrient loadings in breeding pools, contributing to the rapid growth of Cyanobacteria and algae [34]. Consequently, the aquatic photosynthetic organisms (as Chl*a*) in the breeding pools showed differences among seasons. The blooming of aquatic photosynthetic organisms may cause more severe anoxic conditions. However, our results showed that the highest DO levels were observed in summer, suggesting that the photosynthetic organisms in breeding pools were actively photosynthesizing during the daytime to produce O_2_. They were not yet at the stage of eventual die-off and microbial decomposition [35]. Total N, total P, COD_Mn_, and Chl*a* are important indicators of water eutrophication levels. Our results indicated variations in eutrophication levels across different breeding pools and sampling months. All four indicators showed higher values from July to October compared to other sampling months (Figure S2), consistent with our hypothesis. Furthermore, total P and COD_Mn_ levels in the breeding pools were significantly higher in the core region than those at the edge of wetland (Figure S1), which may be due to the lower elevation and plant diversity in the core region. The lower elevation and slower water flow in the center of the wetland led to the deposition of suspended particles and the accumulation of organic matter and nutrients from the surrounding environment. Moreover, a meta-analysis revealed that plant richness positively affected COD and total N removal, and had a marginally positive effect on total P removal [36]. The edge of the wetland had about 70% coverage of vascular plants, including *Osmundastrum cinnamomeum*, *Hemerocallis fulva*, *Hydrangea paniculata*, and *Rhododendron molle*, with a *Sphagnum* carpet, while the dominant vegetation in the core region of the wetland was *Sphagnum*. Therefore, the plants at the wetland edge, with higher diversity, may utilize more nutrients.

### 4.2. Variations in Microbial Communities

Microbial communities exhibited significant variations among different breeding pools, including bacteria, fungi, and protists (Figure 5). Various environmental factors associated with water quality have been demonstrated to influence the community structure of wetland or aquatic microbial communities, including total P concentration, total N concentration, dissolved organic matter (DOM) concentration, and pH [37]. In this study, the Shannon–Wiener and Chao1 indices were lower in the core region compared to those at the edge of the wetland (Figure 5 and Figure S3), contrary to the total P and COD_Mn_ levels. The microbial community, especially the bacterial communities, of Site 2–5 showed clear separation from Site 1, 6, water inlet and outlet according to the NMDS analysis, indicating that the sampling sites were closely associated with water quality and played a critical role in shaping microbial communities. Previous studies reported significant seasonal differences in microbial composition in constructed wetlands or lakes, which could be attributed to variations in physicochemical properties [16,38]. Water temperature, a key seasonal factor, can directly regulate microbial communities and activities of microbial metabolic enzymes [14]. However, contrary to our expectations, microbial diversity did not change significantly across different sampling months (Figure S4), and the spatial variabilities prevailed over seasonal changes shaping microbial communities, according to NMDS analysis. Liu et al. (2023) also reported that the water temperature did not change significantly the total diversity of planktonic bacteria and archaea in a river [13]. Nonetheless, water temperature still played an important role in shaping microbial communities. It was the only factor that collectively influenced bacterial, fungal, and protist communities, as evidenced by the Mantel correlations (Table 1) and microbial relative composition (Figure 6 and Figure S5). Specifically, the abundances of Alveolates were much higher from May to November, in contrast to the abundances of Stramenopiles (Figure S5). Stramenopiles are a eukaryotic supergroup that includes diverse protists, such as diatoms, kelp, and golden algae, while dinoflagellates, apicomplexans, and aplastidial ciliates constitute the supergroup alveolates [39]. The abundances of Proteobacteria were lowest in January and December, contrary to the abundances of Cyanobacteria. These phenomena may be attributed to a selective effect caused by different temperatures, wherein the growth of particularly responsive microbial taxa was promoted by the different temperatures that in turn led to the suppression of other species [40].

The microbial network structures and topological features changed dramatically across breeding pools (Figure 8), indicating a shift in the microbial community [16]. The lowest numbers of nodes and edges were observed in Site 4 (node:138, edge: 638), indicating less complicated links among bacteria, fungi, and protists in the water networks of breeding pools located in the core wetland (Table 2). Conversely, modularity was highest in Site 3 (0.611), decreasing from the core to the edge of the wetland, which also suggested a more disorganized structure in the core area. The higher modularity in the breeding pools within the core wetland can effectively mitigate the impact of species loss on the microbial network [41]. Negative edges occupied a larger proportion than the positive edges in the networks of all breeding pools, indicating higher stability of microbial networks and potential competition among microorganisms [42]. The higher number of module hubs at the edge of the wetland (Site 1, 6, water inlet and outlet) compared to core area (Site 3 and 4) indicated more complex networks, consistent with topological features. Notably, Proteobacteria were the most common module hubs of the microbial network, emphasizing their crucial role in maintaining network stability. The simpler networks of the breeding pools in the core area may be attributed to lower plant community diversity. *Sphagnum*, the dominant moss in the core area of the wetlands, plays a crucial role in regulating microbial structure and activities and acts as a selective factor in shaping microbial communities. The leachates, exudates, and residues of *Sphagnum* accumulated in the breeding pools, especially the core area of the wetland. Hamard et al. (2019) reported that the leachate of *Sphagnum* destabilized microbial networks [43]. Moreover, the complexity of the microbial network was closely related to microbial α-diversity, suggesting that higher microbial α-diversity could contribute to the stability of microbial networks and stronger ecological linkages between microorganisms [12]. Overall, these results suggested that the complexity of networks was subjected to the locations of the breeding pools, and may further impact the function of microbial communities [16].

### 4.3. The Effects of Water Quality on Egg Sacs of Hynobius amjiensis

Egg sacs of *Hynobius amjiensis* were observed in Site 1, 2, 5, and 6, but not in Site 3 and 4. Although the number of egg sacs was highest in Site 5, most of the embryos within the egg sacs did not develop well or died at an early developmental stage (Figure 2). Chen et al. (2016) reported that *H. amjiensis* tended to lay eggs in small pools with cool, clear, and weakly acidic water [8], which is consistent with our survey. The core region of the wetland contained more nutrients, and the level of eutrophication was more severe (Site 3–5) than that at the edge of the wetland (Site 1, 2, and 6). Wetlands can serve as critical hotspots for biogeochemical cycling of nutrients and are a focal point for the accumulation of legacy nutrients within watersheds [44], leading to reduced amounts of DO and the death of aquatic life. The decomposition of algae under anoxic conditions further contributed to the contents of N and P back in the water. Thus, water quality may play a decisive role in the total number and health conditions of egg sacs. In turn, Nagel et al. (2021) assessed the occurrence of the spotted salamander in 71 natural locations and reinforced the utility of egg-mass counts in evaluating vernal-pool habitat quality [45]. Therefore, the conditions of egg sacs can refine our ability to characterize breeding pools and wetlands.

## 5. Conclusions

In this study, six breeding pools of *Hynobius amjiensis* in a tropical *Sphagnum*-dominated wetland were investigated to explore the interactions among egg sac conditions, water quality, and microbial communities. Egg sacs in the breeding pools located at the edge of the wetland developed well, while those in the core area were either absent or died at an early developmental stage. The nutrient concentrations in the breeding pools were significantly higher in the core wetland compared to the marginal area, contrary to the microbial diversity. The relative abundances of dominant phyla or genera were closely related to the water properties. The water microbial communities of the breeding pools showed significant differences between the core wetland and the marginal area due to differing water quality. The complexity of networks was subjected to the location of breeding pools. The less stable networks of the breeding pools in the core wetland may impact the numbers and health of egg sacs. These insights provide support for further investigation of water quality and microbial communities in aquatic ecosystems and their relationships with biodiversity, guiding regulations for wetland conservation and the protection of endangered species.

## Figures and Tables

**Table 1:** Mantel correlations between microbial community and water physicochemical properties. All: all physicochemical properties; Temp: temperature; DO: dissolved oxygen; EC: electric conductivity; NTU: turbidity; COD_Mn_: chemical oxygen demand by potassium permanganate oxidation; Chl*a*: chlorophyll *a*.

Water Properties	Bacteria		Fungi		Protist	
	R	*p* ^1^	R	*p*	R	*p*
All	0.283	<0.001	−0.009	n.s.	0.007	n.s.
pH	0.052	n.s. ^2^	−0.008	n.s.	0.007	n.s.
Temp	0.129	0.007	0.117	0.010	0.068	0.008
DO	0.055	n.s.	−0.018	n.s.	0.019	n.s.
EC	−0.018	n.s.	−0.007	n.s.	−0.017	n.s.
NTU	0.000	n.s.	−0.040	n.s.	−0.009	n.s.
Total N	0.037	n.s.	−0.057	n.s.	−0.025	n.s.
Total P	0.188	0.002	−0.007	n.s.	0.004	n.s.
COD_Mn_	0.216	<0.001	0.035	n.s.	0.044	n.s.
Chl*a*	0.335	<0.001	0.038	n.s.	0.025	n.s.

^1^ *p* was derived from 9999 permutations. ^2^ n.s. indicates not significant (*p* ≥ 0.05).

**Table 2:** The topological properties of co-occurrence networks in water in different sampling sites for all months.

Topological Properties	Inlet	Site 1	Site 2	Site 3	Site 4	Site 5	Site 6	Outlet
Numbers of modules (containing more than 5 nodes)	7	4	7	4	5	7	5	6
Number of nodes	463	594	249	240	138	189	186	413
Number of edges	5466	15,270	2110	2508	638	1137	1351	7548
Positive edges (%)	28.8	31.8	20.4	29.5	22.6	25.9	25.7	34.8
Negative edges (%)	71.2	68.2	79.6	70.5	77.4	74.1	74.3	65.2
Average degree	23.61	51.41	16.95	20.90	9.25	12.03	14.53	36.55
Modularity	0.427	0.391	0.439	0.611	0.428	0.496	0.425	0.396
Average betweenness centrality	441.3	460.5	234.1	238.4	159.8	186.2	192.3	351.9
Average closeness centrality	0.349	0.399	0.359	0.338	0.311	0.348	0.334	0.379
Average clustering coefficient	0.390	0.471	0.448	0.565	0.399	0.453	0.469	0.491
Average path distance	2.910	2.553	2.950	2.995	3.333	3.068	3.079	2.708

## Data Availability

All data generated or analyzed during this study are included in this published article. Sequences were submitted to the Genome Sequence Archive (GSA) database under accession number CRA016685 and CRA016718.

## Supplementary Materials

The following supporting information can be downloaded at: https://www.mdpi.com/article/10.3390/microorganisms12071344/s1, Illumina high-throughput sequencing methods; Table S1: The characteristics of six breeding pools; Table S2: The potential keystone taxa in network according to connectivity within module (*Zi*) and connectivity among module (*Pi*); Figure S1: Variations of total N (A), total P (B), chemical oxygen demand by potassium permanganate oxidation (C) and chlorophyll a (D) in water of different breeding pools with all sampling months; Figure S2: Variations of total N (A), total P (B), chemical oxygen demand by potassium permanganate oxidation (C), and dissolved oxygen (D) in water of different sampling months with all breeding pools; Figure S3: Boxplots of α-diversity as revealed by Chao1 index for bacterial (A), fungal (B), and protist (C) communities in the water samples of different breeding pools with all sampling months; Figure S4: Boxplots of α-diversity as revealed by Chao1 and Shannon–Wiener index for bacterial (A,D), fungal (B,E), and protist (C,F) communities in the water samples of different sampling months with all breeding pools; Figure S5: Relative abundances of bacteria (A,B), fungi (C,D), and protists (E,F) among different sampling sites or months at the phylum level.
