# Supplementary material for: Spatial and Seasonal Changes in Microbial Community of Hynobius amjiensis Breeding Pools in a Sphagnum-Dominated Peatland
Source: Microorganisms. 2024 Jun 30;12(7):1344. doi: 10.3390/microorganisms12071344 (PMC11279080; doi:10.3390/microorganisms12071344)
Supplement: Supplementary file 1 [file microorganisms-12-01344-s001.zip › microorganisms-3055810-supplementary.pdf]

Supporting information

Spatial and seasonal changes in microbial community of *Hynobius amjiensis* breeding pools in a *Sphagnum*-dominated peatland

Meng-Jie Yu [1], Xian-Ting Wang [2], Ting Wang [1], Wei-Quan Huang [1], Ze-Dong Lang [2], Jia-Peng Wang [3,4,*], and Yu-Huan Wu [1,*]

[1] College of Life and Environmental Sciences, Hangzhou Normal University, Hangzhou, China

[2] Zhejiang *Hynobius amjiensis* Nature Reserve Management Office, Huzhou, China

[3] School of Information Science and Technology, Hangzhou Normal University, Hangzhou, China

[4] Zhejiang Provincial Key Laboratory of Urban Wetlands and Regional Change, Hangzhou, China

[*] **Corresponding authors**:

Yu-Huan Wu; **E-mail**: yuhuanwu@hznu.edu.cn

Jia-Peng Wang; **E-mail**: wangjiapeng@hznu.edu.cn

Illumina high-throughput sequencing

Sample-specific 7-bp barcodes were incorporated into the primers for multiplex sequencing. Each sample was amplified in a 25-μL system containing 5 μL of buffer (5×), 0.25 μL of Fast pfu DNA Polymerase (5 U/μL), 2 μL of dNTPs (2.5 mM), 1 μL of each primer (10 μM), 1 μL of DNA sample, and 14.75 μL of ddH_2O. The thermal cycling for 16S rRNA gene consisted of 98 °C for 3 min, then 26 cycles of 98 °C for 30 s, 52 °C for 30 s, 72 °C for 45 s, and a final extension of 72 °C for 5 min. The thermal cycling for 18S rRNA gene consisted of 98 °C for 3 min, then 33 cycles of 98 °C for 30 s, 46 °C for 30 s, 72 °C for 45 s, and a final extension of 72 °C for 5 min. Amplified samples were purified with VAHTS® DNA Clean Beads (Vazyme, China) and quantified using the Quant-iT™ PicoGreen™ dsDNA Assay Kit (Invitrogen, USA). The amplicons were pooled in equal amounts, pair-end 2×250 bp sequencing was performed using the Illlumina NovaSeq platform with NovaSeq™ 6000 S Prime Reagent Kit (Illlumina USA).

Table S1. The characteristics of six breeding pools.

Breeding pools	Site 1	Site 2	Site 3	Site 4	Site 5	Site 6
Area (m^2)	0.30	2.00	1.77	0.24	0.77	0.49
Minimum water depth (m)	0.76	1.57	0.99	0.59	0.86	0.6
Maximum water depth (m)	0.82	1.85	1.23	0.79	1.5	0.82
Number of egg sacs	1	3	0	0	14	2

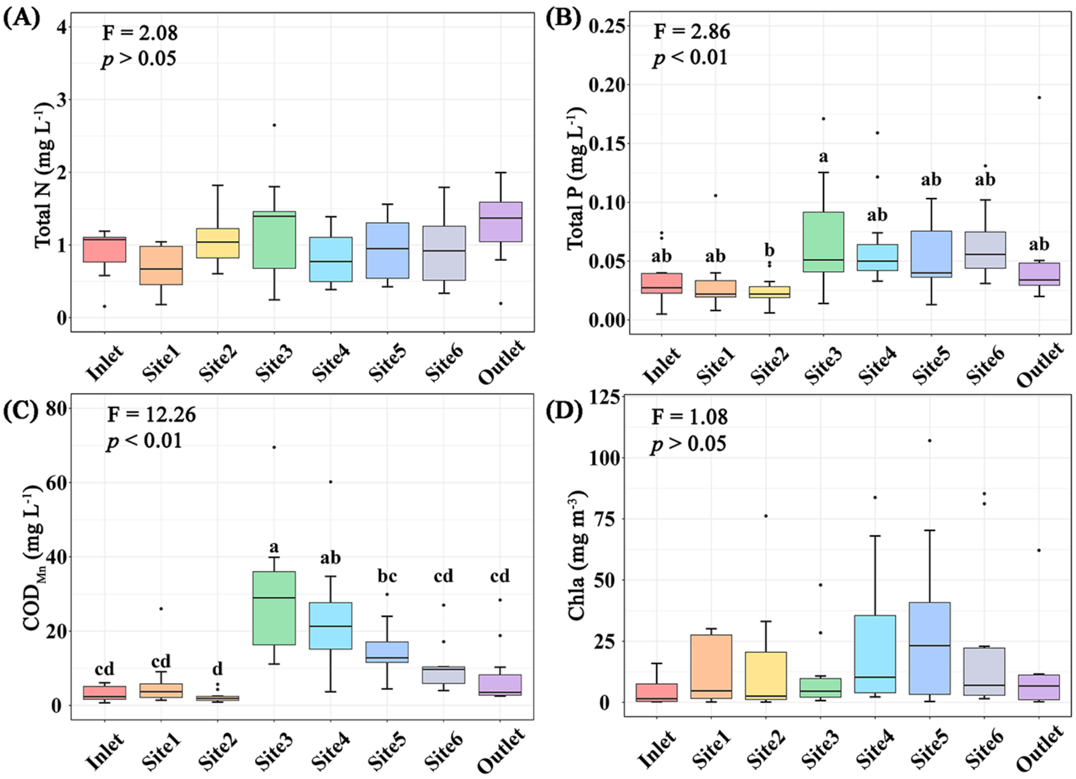

Figure S1. Variations of total N (A), total P (B), chemical oxygen demand by potassium permanganate oxidation (C) and chlorophyll *a* (D) in water of different breeding pools with all sampling months. Boxes are bounded on the first and third quartiles, divided by median lines. Black dots represent outlying data. Boxes with different lower-case letters are significantly different ($p < 0.05$) using Tukey HSD post-doc test.

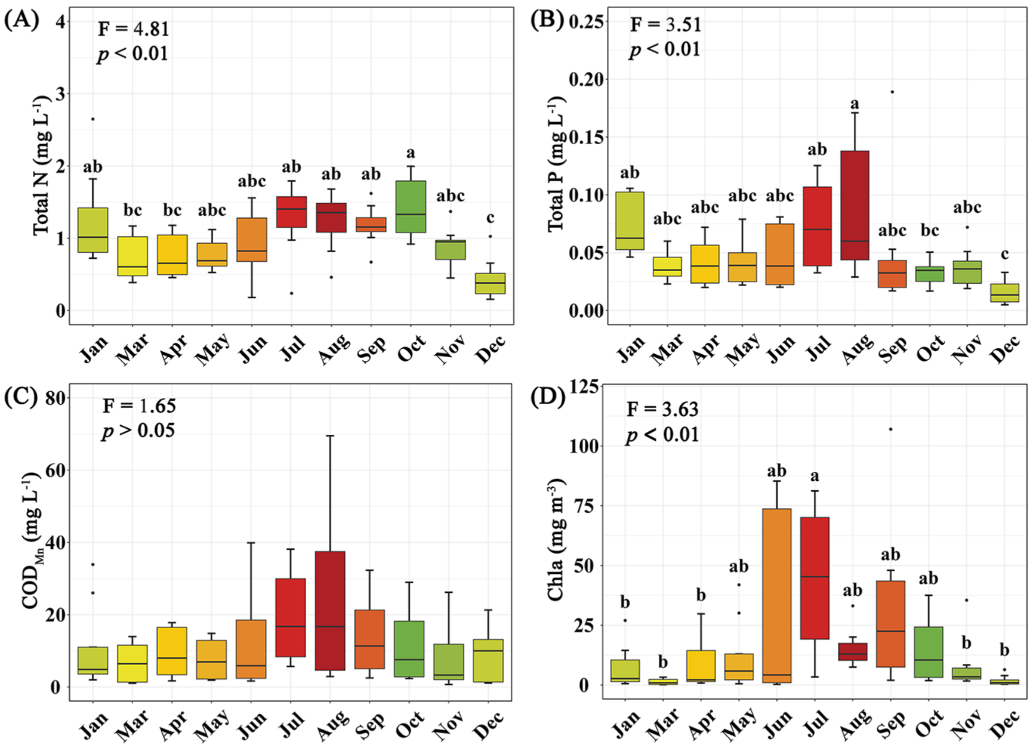

Figure S2. Variations of total N (A), total P (B), chemical oxygen demand by potassium permanganate oxidation (C) and dissolved oxygen (D) in water of different sampling months with all breeding pools. Boxes are bounded on the first and third quartiles, divided by median lines. Black dots represent outlying data. Boxes with different lower-case letters are significantly different ($p < 0.05$) using Tukey HSD post-doc test.

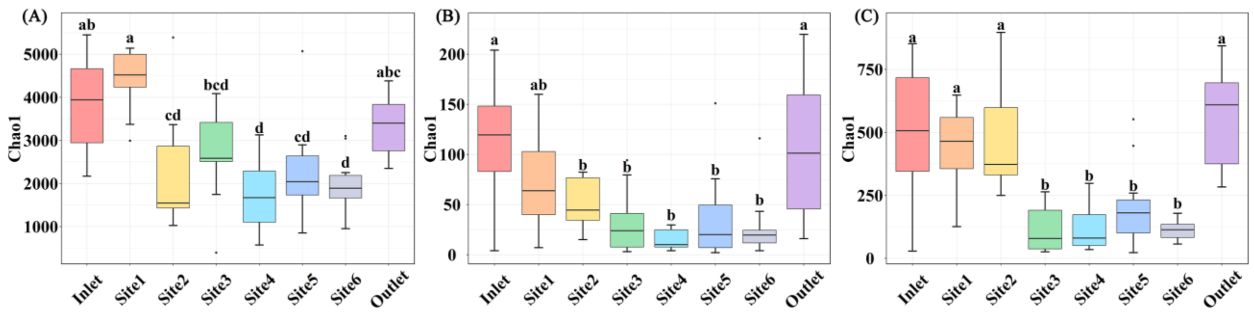

Figure S3. Boxplots of α-diversity as revealed by Chao1 index for bacterial (A), fungal (B) and protist (C) communities in the water samples of different breeding pools with all sampling months. Boxes are bounded on the first and third quartiles, divided by median lines. Black dots represent outlying data. Boxes with different lower-case letters are significantly different ($p < 0.05$) using Tukey HSD post-doc test.

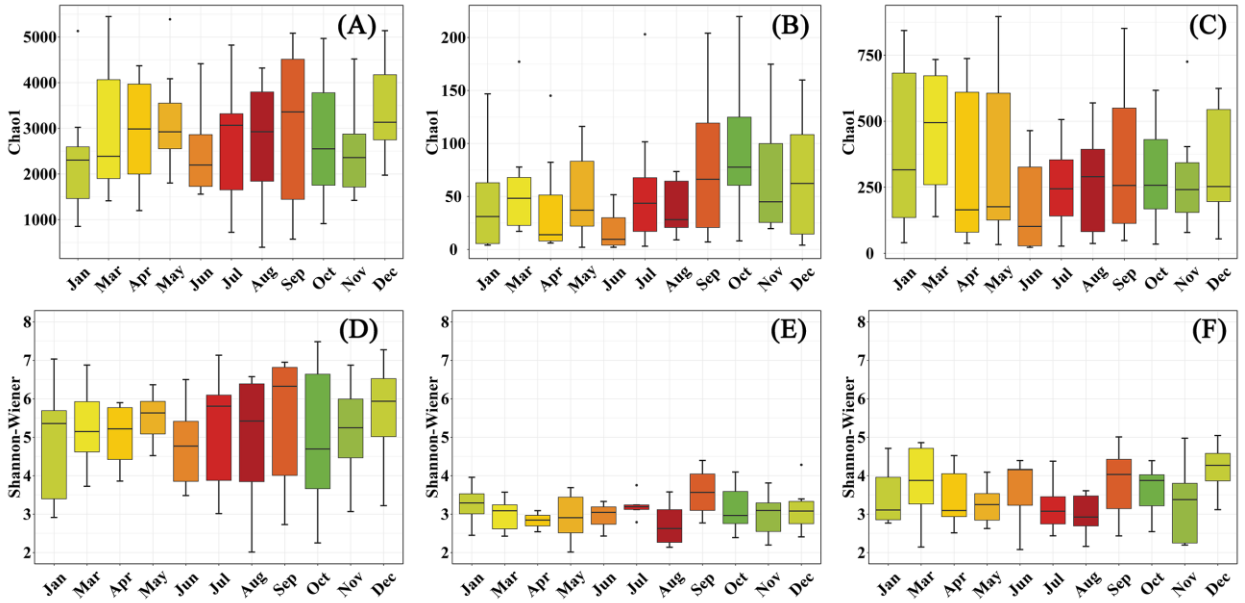

Figure S4. Boxplots of α-diversity as revealed by Chao1 and Shannon-Wiener index for bacterial (A and D), fungal (B and E) and protist (C and F) communities in the water samples of different sampling months with all breeding pools. Boxes are bounded on the first and third quartiles, divided by median lines. Black dots represent outlying data. Boxes with different lower-case letters are significantly different ($p < 0.05$) using Tukey HSD post-doc test.

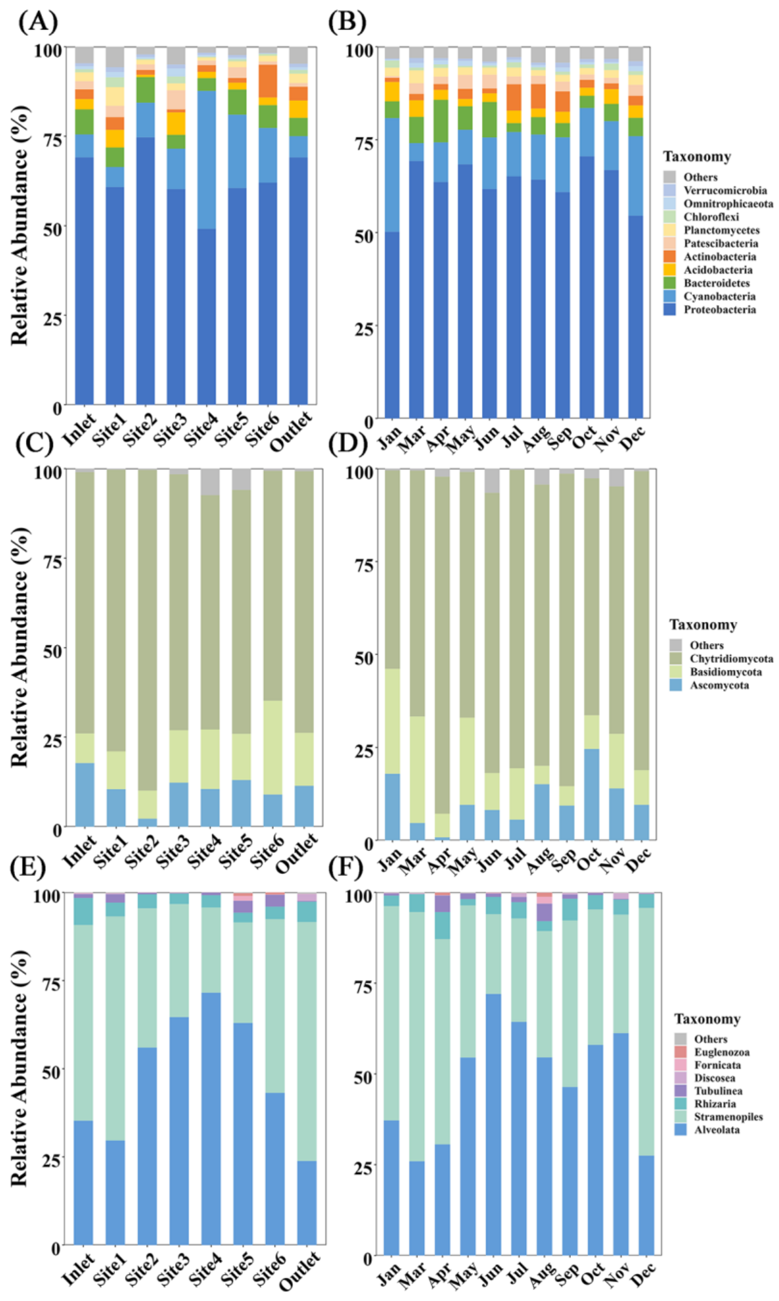

Figure S5. Relative abundances of bacteria (A and B), fungi (C and D) and protist (E and F) among different sampling sites or months at the phylum level.

Table S2. The potential keystone taxa in network according to connectivity within module (*Zi*) and connectivity among module (*Pi*). All nodes were classified into four categories based on *Zi* and *Pi* values: network hubs (*Zi* ≥ 2.5 and *Pi* ≥ 0.6), module hubs (*Zi* ≥ 2.5 and *Pi* < 0.62), connectors (*Zi* < 2.5 and *Pi* ≥ 0.6) and peripherals (*Zi* < 2.5 and Pi < 0.6).

Site	Number of connectors	Number of module hubs	Module hubs
**Inlet**	100	5	**Protist**; TSAR; Stramenopiles; Gyrista; Chrysophyceae; Ochromonadales*; Ochromonadales_clade-XII; Ochromonadales_clade-XII_X; Ochromonadales_clade-XII_X*_sp.
			**Protist**; Alveolata; Ciliophora; Oligohymenophorea; *Hymenostomatia; Tetrahymenida*
			**Bacteria**; Proteobacteria; Gammaproteobacteria; Pseudomonadales; *Moraxellaceae; uncultured; metagenome*
			**Bacteria**; Proteobacteria; Alphaproteobacteria; uncultured*; uncultured; uncultured; metagenome*
			**Bacteria**; Proteobacteria; Gammaproteobacteria; Betaproteobacteriales; *Burkholderiaceae; unclassified_Burkholderiaceae; unclassified_Burkholderiaceae*
**Site 1**	91	3	**Bacteria**; Proteobacteria; Gammaproteobacteria; Betaproteobacteriales; *Rhodocyclaceae; Denitratisoma; unclassified_Denitratisoma*
			**Bacteria**; Patescibacteria; CPR2; CPR2; *CPR2; CPR2; uncultured_Clostridium*
			**Bacteria**; Proteobacteria; Deltaproteobacteria; Desulfuromonadales; *Geobacteraceae; Geobacter; unclassified_Geobacter*
**Site 2**	65	1	**Bacteria**; Proteobacteria; Alphaproteobacteria; Rhizobiales; *Beijerinckiaceae; Roseiarcus; uncultured_bacterium*
**Site 3**	20	0	
**Site 4**	53	0	
**Site 5**	63	1	**Bacteria**; Proteobacteria; Alphaproteobacteria; Rhizobiales; Rhizobiaceae; Allorhizobium-Neorhizobium-Pararhizobium-Rhizobium; unclassified_Allorhizobium-Neorhizobium-Pararhizobium-Rhizobium
**Site 6**	36	2	**Bacteria**; Actinobacteria; Actinobacteria; Micrococcales; *Microbacteriaceae; unclassified_Microbacteriaceae; unclassified_Microbacteriaceae*
			**Bacteria**; Chloroflexi; KD4-96; KD4-96; *KD4-96; KD4-96; unclassified_KD4-96*
**Outlet**	68	3	**Bacteria**; Proteobacteria; Alphaproteobacteria; Rhizobiales; *Xanthobacteraceae; Pseudolabrys; unclassified_Pseudolabrys*
			**Bacteria**; Acidobacteria; Subgroup_22; Subgroup_22; *Subgroup_22; Subgroup_22; uncultured_Holophaga*
			**Bacteria**; Proteobacteria; Deltaproteobacteria; Bdellovibrionales; *Bacteriovoracaceae; uncultured; metagenome*
